# Supplementary material for: Wading through Molasses: A qualitative examination of the experiences, perceptions, attitudes, and knowledge of Australian medical practitioners regarding medical billing
Source: PLoS One. 2022 Jan 21;17(1):e0262211. doi: 10.1371/journal.pone.0262211 (PMC8782346; doi:10.1371/journal.pone.0262211)
Supplement: S1 File — (PDF) [file pone.0262211.s001.pdf]

UNIVERSITY OF TECHNOLOGY SYDNEY

PARTICIPANT INFORMATION SHEET (Phase 2)

PROJECT TITLE

Claiming and compliance under the Medicare Benefits Schedule (MBS): a critical examination of attitudes, experiences, perceptions and knowledge of medical practitioners, UTS HREC REF NO. 2014000060.

WHO IS DOING THE RESEARCH?

My name is Margaret Faux and I am a PhD candidate at UTS. My supervisors are Jon Wardle and Jon Adams.

WHAT IS THIS RESEARCH ABOUT?

The aim of my research is to examine the experiences and perceptions of medical practitioners as they interact with Medicare and claim MBS reimbursements. The research also aims to identify any perceived barriers to compliance and to explore possible solutions to problems and deficiencies identified by participants.

IF I SAY YES, WHAT WILL IT INVOLVE?

I will ask you to participate in one face to face interview of between 30 minutes and one hour. You can choose the location and time of the interview.

ARE THERE ANY RISKS/INCONVENIENCE?

There are very few if any risks because the research has been carefully designed. Your privacy is of the highest importance and the data collected will be de-identified prior to being analysed and/or published.

WHY HAVE I BEEN ASKED?

You have been asked to participate because you are a medical practitioner who claims MBS reimbursements.

DO I HAVE TO SAY YES?

You don't have to say yes.

WHAT WILL HAPPEN IF I SAY NO?

Nothing. I will thank you for your time so far and won't contact you about this research again.

IF I SAY YES, CAN I CHANGE MY MIND LATER?

You can change your mind at any time and you don't have to say why. I will thank you for your time so far and won't contact you about this research again.

WHAT IF I HAVE CONCERNS OR A COMPLAINT?

If you have concerns about the research that you think I or my supervisor can help you with, please feel free to contact us on:

Margaret Faux: 0414 600 073
Margaret.A.Faux@student.uts.edu.au
Jon Wardle: Jon.Wardle@uts.edu.au
Jon Adams: Jon.Adams@uts.edu.au

If you would like to talk to someone who is not connected with the research, you may contact the Research Ethics Officer on 02 9514 9772, and quote this number UTS HREC REF NO. 2014000060.
