# Supplementary material for: Wading through Molasses: A qualitative examination of the experiences, perceptions, attitudes, and knowledge of Australian medical practitioners regarding medical billing
Source: PLoS One. 2022 Jan 21;17(1):e0262211. doi: 10.1371/journal.pone.0262211 (PMC8782346; doi:10.1371/journal.pone.0262211)
Supplement: S2 File — (PDF) [file pone.0262211.s002.pdf]

UNIVERSITY OF TECHNOLOGY SYDNEY

***Participant Consent Form (Phase 2)***

I ____________________ *(participant's name)* agree to participate in the research project;

*Claiming and compliance under the Medicare Benefits Schedule (MBS): a critical examination of attitudes, experiences, perceptions and knowledge of medical practitioners*, UTS HREC REF NO. 2014000060.

The project is being conducted by Margaret Faux, email: Margaret.A.Faux@student.uts.edu.au telephone: 0414 600 073 of the University of Technology, Sydney, for her PhD.

I understand that the purpose of this study is to examine the experiences and perceptions of medical practitioners as they interact with Medicare and claim MBS reimbursements. The research also aims to identify any perceived barriers to compliance and to explore possible solutions to problems and deficiencies identified by participants.

I understand that I have been asked to participate in this research because I am a medical practitioner who claims MBS reimbursements in my daily work and that my participation in this research will involve between 30 minutes and one hour of my time being interviewed. There are no foreseeable risks to me above the risks of everyday living.

I am aware that I can contact Margaret Faux or her supervisors, Jon Wardle or Jon Adams, if I have any concerns about the research. I also understand that I am free to withdraw my participation from this research project at any time I wish, without consequences, and without giving a reason.

I agree that Margaret Faux has answered all my questions fully and clearly.

I agree that the research data gathered from this project may be published in a form that does not identify me in any way.

________________________________________ ____/____/____

Signature (participant)

________________________________________ ____/____/____

Signature (researcher or delegate)

**NOTE:**

This study has been approved by the University of Technology, Sydney Human Research Ethics Committee. If you have any complaints or reservations about any aspect of your participation in this research which you cannot resolve with the researcher, you may contact the Ethics Committee through the Research Ethics Officer (ph: +61 2 9514 9772 Research.Ethics@uts.edu.au) and quote the UTS HREC reference number. Any complaint you make will be treated in confidence and investigated fully and you will be informed of the outcome.
