# Supplementary material for: Wading through Molasses: A qualitative examination of the experiences, perceptions, attitudes, and knowledge of Australian medical practitioners regarding medical billing
Source: PLoS One. 2022 Jan 21;17(1):e0262211. doi: 10.1371/journal.pone.0262211 (PMC8782346; doi:10.1371/journal.pone.0262211)
Supplement: S3 File — (PDF) [file pone.0262211.s003.pdf]

Appendix 1 - Qualitative Interview Question Guide

1. Do you recall your first experience billing your first MBS item? Can you tell me about that?
2. How did you initially learn to navigate the MBS?
3. Do you feel that your education, including undergraduate, post graduate and CME, has adequately equipped and informed you in relation to your MBS compliance obligations? If the answer identifies deficiencies: What do you perceive as being the nature of the deficiencies? Do you have any suggestions as to how the deficiencies might be addressed?
4. Have you ever encountered any problems or difficulties claiming MBS reimbursements that affected you personally? Have you ever encountered any problems or difficulties that affected your patients?
5. What do you understand as being the purpose of Medicare and having a provider number? How do you enact this understanding in day-to-day practice?
6. What do you view as your rights, obligations and responsibilities in relation to Medicare and the MBS?
7. Are you aware of possible repercussions for non-compliance with the MBS? Do you feel any concern about possible repercussions for non-compliance?
8. Do you manage your own MBS claims or do you outsource or delegate this task to third parties (such as practice managers or billing services)? If yes: What benefits do you perceive from doing this? What potential risks do you perceive from doing this?
9. What level of detail are you able to recall about your claiming patterns and practices?
10. Do you perceive differences between bulk billing transactions and other transactions? In what circumstances do you perceive you are able to charge additional fees to your patients?
11. Do you perceive any external pressure in relation to your claiming? What is the basis for this perception? (Note whether these are different in differing practice types e.g. corporate v. solo practice)
12. What do you perceive as being the relationship between your compliance obligations and patient care? Do you perceive that your billing patterns may impact patient care?
13. What types of support do you seek in relation to MBS billing? How often do you seek support in relation to your claiming? What do you perceive as being the quality of the support you receive? Where are you most likely to turn to for support in this area?
14. Do you perceive barriers or issues that prevent you from seeking or gaining assistance?
15. Do you perceive that your MBS claiming is compliant with current standards in your current practice setting? Why? Why not? Would you feel confident if you were audited by Medicare or the PSR? Why? Why not?
16. What do you understand about the aftercare claiming rules?
17. What do you understand as being the patient’s role in a bulk billing transaction?
18. What do you understand about the rules around valid referrals?
19. What do you understand about the provider number rules and which one to use when?
20. What do you understand about the rules concerning the charging of Veterans, serving members and WC/TP patients?
21. What are your perceptions in relation to your claiming patterns and your responsibility for the national health budget?
