# Supplementary material for: Wading through Molasses: A qualitative examination of the experiences, perceptions, attitudes, and knowledge of Australian medical practitioners regarding medical billing
Source: PLoS One. 2022 Jan 21;17(1):e0262211. doi: 10.1371/journal.pone.0262211 (PMC8782346; doi:10.1371/journal.pone.0262211)
Supplement: Attachment — Submitted filename: Response to reviewers_WTM_letter.docx [file pone.0262211.s004.docx]

Margaret Faux

PhD Candidate and Corresponding Author

Faculty of Health

University of Technology Sydney

18 September 2021

Editor

*PloS One*

Dear Editors,

**RE: PONE-D-20-16346R1
Wading through Molasses: A qualitative examination of the experiences, perceptions, attitudes, and knowledge of Australian medical practitioners regarding medical billing.**

Thank you for the opportunity to revise the above paper. We have addressed each of the reviewer comments below. We have also attached the revised paper with tracked changes.

We trust that we have satisfactorily addressed the minor revisions requested, and look forward to hearing from you shortly.

Yours sincerely

Margaret Faux (on behalf of all authors)

***REVIEWER #2:***

1) There is still a significant amount of jargon in this revised manuscript. Please consider defining all of the terms (such as bulk billing and gap), or add a glossary of terms for the reader (or both). These terms are not in common use outside of Australia.

*We thank the reviewer for their comments and have made significant changes to address this issue. We have not added a glossary because by removing a considerable amount of Australia specific jargon (described below), there were only two words requiring definition (bulk billing and gap), both of which we have explained and addressed with the following* ***highlighted*** *amendments.*

1. P 11 para 2 – added additional bracketed text as follows:

“…when I did my GP training we had a block of training prior to our very first day on the job…we basically just learnt you know your 23 and 36 item number **[common time based attendances]**^11^... there would have been question and answer time, but we hadn’t practised yet so we wouldn’t really have known what questions to ask.” (GP1)

2	P 12 para 4, we have added the following clear explanation of bulk billing (while also retaining further explanation in the reference) as well as providing a simple explanation of a gap, as follows:

When participants were asked detailed questions about fundamental legal requirements to bill correctly, their levels of literacy were variable and some were confused in important areas, such as when it is permissible to charge a gap and what bulk billing was. **Bulk billing is a common term in Australia, describing a transaction for a medical service wherein the patient does not pay any money because the medical practitioner chooses to accept the amount of the available government subsidy for that service.^12^ The term ‘gap’ in the Australian context refers to a patient out-of-pocket payment which in many countries is described as a co-payment.**

3	P 14 para 2, we have made the following highlighted additions to explain another element of Australian medical billing more explicitly:

The majority of participants were also unclear about fundamental billing requirements. **In Australia’s gatekeeper model health system, patients usually require a valid referral from a general practitioner before seeking more specialised care. However, most participants did not know what constituted a valid referral. Other very basic requirements to bill correctly were also poorly understood by most participants such as specific rules around billing eligible war veterans, and whether any patient has to sign a form when the medical practitioner bulk bills the patient.**

4	P 23, last para – we have removed the following Australia specific highlighted text:

The data also suggest the current ‘rules’ of medical billing are confusing, and medical practitioners are struggling to understand and apply them in daily practice. **~~Available evidence also suggests that recent Australian Government initiatives such as the MBS Review Taskforce (MBSRT)~~^~~18~~^ ~~may be exacerbating these problems by making it difficult for medical practitioners to keep pace with the Australian Governments’ frenetic law making.~~^~~2~~^**

5.	Page 25 last para and page 26 first two paras – we have deleted the following Australia specific content completely:

**~~Another potential impact on public hospitals caused by disparate Federal and State agencies, is that well-intentioned, but siloed initiatives such as the MBSRT, may cause disruptions to legitimate revenue streams for State Governments when clinical code sets diverge. In a recent example, changes to the MBS colonoscopy items recommended by the MBSRT were introduced on 1 November 2019. These changes turned one MBS item number into seven, none of which matched the Australian Classification of Health Intervention Codes (ACHI).~~^~~21~~^ ~~ACHI codes have a number of purposes including to code private patient encounters in public hospitals and when the codes submitted by a medical practitioner using the MBS do not match the codes submitted by the hospital using ACHI, for the same episode of care, Private Health Insurers may reject or delay payment, incorrectly assuming either the hospital or the medical practitioner has submitted a non-compliant bill.~~**

**~~ACHI codes are updated biennially and despite being derived from the MBS, often differ from the MBS for the very same service, because ACHI represent different concepts intended for different use cases and are the responsibility of the Independent Hospitals Pricing Authority (IHPA).~~^~~21~~^ ~~IHPA is focussed on hospital morbidity and mortality, whereas members of the MBSRT were predominantly medical practitioners, who were understandably focussed on writing MBS service descriptions that meant something to them. In addition to impacting State Government revenue streams, this increasing divergence in our national clinical classifications and code sets may ultimately hamper full implementation of Australia’s National Digital Health Strategy, which envisions standard semantic interoperability.~~^~~22~~^ ~~To prevent this, it will be critical to ensure future code committees include individuals with the necessary skills to understand e-enabled health environments and work collaboratively aligning their codes with each other and with additional international codes already in use in Australia, such as SNOMED-CT and ICD-10AM.~~**

6.	Page 29, second para, we have removed the below highlighted text to align the changes described above.

Non-compliant medical billing under Australia’s Medicare scheme is a nuanced phenomenon that may be far more complex than previously thought. Therefore, many of the current punitive, post payment audit initiatives of the government are unlikely to succeed. **~~such as trying to nudge medical practitioners into compliance with non-existent or incomprehensible rules they have never been taught and do not understand.~~**

7.	Page 29, last para – we have made the below highlighted changes to align the changes described above.

Strategies to address the barriers and deficiencies identified by participants in this study will require a multi-pronged approach which may include the development of clear, legally binding medical billing rules, nationally consistent, accurate and accessible education, and structural reform to tighten**~~ing~~** and align**~~ment~~** **~~of~~** the underlying regulatory framework. **~~including aligning national code sets.~~**

8.	Page 30 second para, we have removed the acknowledgment of Heather Grain because we have removed all reference to the Australia specific areas which we discussed with her.

**~~The authors acknowledge the contribution of Heather Grain: Health Informatician, Clinical Coder, Health Information Manager, Digital Health Expert, for her input into Australia’s digital health environment and ACHI codes.~~** The authors **~~also~~** wish to thank the medical practitioners who shared their experiences with medical billing and Medicare. The project received no funding.

2) You may want to consider a table highlighting your significant themes for the reader.

*We thank the reviewer for this suggestion and have added a table (****Table 1****), which occupies the whole of page 10, displaying raw data and its analysis to themes. The addition of the table also necessitated moving two large quotes from the body of the paper and placing them in the table. These are highlighted in the table, which is copied in full below:*

**Table 1**

| **Raw Data** | **Theme** |
| --- | --- |
| [Interviewer asked SMO7 if education at various levels adequately equipped him to bill correctly] Not at all. It is purely through by necessity to understand it oneself and to understand the vagaries not only of billing, but how it works in the context of the staff specialist or ward arrangements, which are quite complex. [interviewer: ‘any education on that either?’] No zero. Zip. | Inadequate induction |
| Bulk billing, I understand is where whatever Medicare says, so if … I treat the patient for say keeping on breathing machine let us say. Government says you can earn $50 a day for doing that and bulk bill would be if I say okay give me $50. If I charge $60, then I have charged a gap. [interviewer: when can you do that? SMO12 replied] No idea. | Poor legal literacy |
| [interviewer]…so when it goes off into accounts, how confident are you about what happens next? [SMO14] I am confident because as the director, I have explored that, my colleagues would be somewhat less confident. [interviewer] With item numbers…? [SMO14] No just total numbers. Just money. Could have been anything. So, in fact, in reality I have no idea. [interviewer] So…you have got an idea of the total dollar amount that is billed, do you have an idea of the actual item numbers? [SMO14] No, not at all, not a jot, not one single solitary scintilla. | Absence of reliable advice and support |
| [GP3] We have a practice manager and we have asked her to contact Medicare about some…uncertain issues regarding Medicare…and she will get five different answers from five different people that she rings…that is a regular experience and I say “…there’s no point in ringing Medicare about this” because I do not know who she is speaking to. I do not know whether she is speaking to a manager…or somebody who has recently started in Medicare who does not have much experience…and is just reading from one part of the manual but doesn’t know the other parts…we’ve always had that experience if you ring up…the most recent example…charging through Medicare for overseas travel…she has spoken to several different people and received different answers from each one. |  |
| [GP2] I probably underbill…I’m just going to do what I know is safe. | Fear and Deference |
| [GP4] The threat of audit kind of hangs over… |  |
| **[SMO7] I do not order a lot of blood tests. I do not order a lot of scans. I am very interested in…evidence base, I am interested in doing what is needed, I try not to pander to anxiety, it’s very difficult, it is much easier to give in and just order a million tests…It is an impost on the national health, so I think there is a responsibility.** |  |
| **[GP8] Sending some more resources …for educating the doctors, by various means be it sending them letters like case examples, emails, having some conferences around, you know, correct Medicare billing etc and educating doctors the implications of incorrect charging particularly over-servicing and fraud, I think that is very important. Doctors just learn from their colleagues and others, you know, we are hearing stories, it is not something they are actively involved in, so there should be an education process and may be even attaching some category points…if the doctors understood Medicare and I think that is very important. The system is there but is not enough education about it.** | Unmet opportunities for improvement |

***INTERNAL REVIEW:***

1) The paragraph ‘Government maladministration’ includes a rather strong criticism towards the Australian government. We recommend that you consider toning down the language of this paragraph, ensuring that all statements made are adequately related to and supported by the data showed in the manuscript. For example, the statement “It would appear the Australian Government is either unable or unwilling to explain the very medical billing laws it promulgates […]” appears to be too speculative and may attract undue external criticism.

*We thank the reviewer for their comments and have made the following* ***highlighted*** *amendments and deletions throughout the manuscript, to soften the language as suggested. We did not mean to suggest that maladministration was intentional or deliberate, but that factors including poor resourcing and support were contributing to the problem.*

1. P 5 para 1, we have made the following changes:

This is largely because the problem is not what can be seen, but what cannot. Lax regulation, **poor** **~~government~~** **~~mal~~**administration, system complexity and the fact that…

2.	P 19 para 2, we have made the following changes:

The participant was subjected to what appears to have been a mishandled audit by Medicare, who **~~did not~~** **appeared to have misunderstood** the operation of the rule, which at all relevant times was clearly described in the MBS. As a result of the audit and Medicare’s failure to explain to the SMO what she did wrong (which **~~appears to~~** **may** have **~~in fact~~** been nothing), the SMO changed her billing behaviour and is now billing incorrectly and costing Australian taxpayers more.

3.	Page 27, the Title ‘Government maladministration’ has been replaced by **‘Inadequate government support’**

4.	Page 27 last para and page 28 first two paras, we have made the following changes:

**Participants reported that** **~~It was apparent that~~** **the** “the blind leading the blind” method by which medical billing information is disseminated may be perpetuating errors and myths. Further, the consistency in the experiences of the wide cross section of participants in this study supports a finding that extremely low levels of legal literacy in relation to medical billing **~~is fact rather than hyperbole~~**, **~~and there~~** may be **creating** a vortex of misinformation contributing to health system leakage.

Further, the data suggest that **~~apparent maladministration~~** **a lack of administrative resources and support provided** by the Australian Government **~~appears to~~** **may** have left medical practitioners with no place to go for legally accurate, reliable advice, meaning that despite due diligence, a medical practitioner may still fall foul of the law. In one case, a participant who **described correct billing practices, appears to have been led ~~had been billing correctly~~**, **~~was effectively led~~** into incorrect billing by the Australian Government who **may** not **have the appropriate resources to provide accurate interpretations of** **~~appeared~~** **~~to have understood~~** its own rules **to practitioners.**

**~~It would appear the Australian Government is either unable or unwilling to explain the very medical billing laws it promulgates, and as such, courts and other authorities must give due consideration to the veracity of any submission made by a medical practitioner under investigation for incorrect billing, relating to ignorance of relevant requirements or the potential impact of third parties on their billing.~~** The participants of this study were clear that expecting medical practitioners to comply with complex **and mercurial** billing laws without relevant skills or training was unrealistic. Moreover, it is suggested that denying medical practitioners access to clear, reliable advice and **~~support~~** **training** prior to imposing sometimes very serious sanctions is indefensible and may be inconsistent with common law principles of natural justice.^24^

2) Please include additional information regarding the interview guides used in the study and ensure that you have provided sufficient details that others could replicate the analyses. For instance, if you developed an interview guide as part of this study and it is not under a copyright more restrictive than CC-BY, please include a copy, in both the original language and English, as Supporting Information.

*We thank the reviewer for this suggestion.*

On page 8, last para, we have added the below text and have attached the question guide to our submission as Appendix 1:

The interviews were semi-structured, with a question sheet used to loosely guide questioning. **A copy of the question guide is shown as Appendix 1.**

*In addition to all of the above changes, we have made minor grammatical changes which are shown on the tracked version of the article, and have adjusted the reference list to align the changes made. This has reduced the number of references to 21.*
